# Exploring Reproductive Timing in Olive Tree: Male Meiosis and Anthesis Events

**DOI:** 10.3390/plants14162522

**Published:** 2025-08-13

**Authors:** Emma Tedeschini, Fabio Orlandi, Marco Fornaciari

**Affiliations:** 1 Department of Agricultural, Food and Environmental Sciences, University of Perugia, Borgo XX Giugno 74, 06121 Perugia, Italy; emma.tedeschini@unipg.it; 2 Department of Civil and Environmental Engineering, University of Perugia, Borgo XX Giugno 74, 06121 Perugia, Italy; marco.fornaciaridapassano@unipg.it

**Keywords:** olive, phenology, pollination, male meiosis, climate changes

## Abstract

The timing of male meiosis plays a pivotal role in ensuring successful pollination and may represent a critical window during which environmental stressors can significantly impact reproductive outcomes. In anemophilous plants, both the quantity of microspores produced and the development of viable pollen are particularly susceptible to external influences, such as fluctuating climatic conditions. This study undertakes a comprehensive analysis of reproductive features, focusing on the meiotic events of male gametogenesis and the phenological phases from the onset to full flowering in olive from central Italy. Utilizing a robust 11-year database (2012–2022), the research incorporates detailed micro- and macro-phenological observations alongside systematic pollen monitoring. The temporal regulation of male meiosis directs the phenological plasticity of the olive tree (*Olea europaea* L.) by transforming it into maladaptive phenological plasticity, effectively making the plant insensitive to thermal changes. This remarkable physiological trait underscores the resilience of this woody species to climate change. The results obtained will help to elucidate the interaction between climatic factors and reproductive dynamics, contributing valuable insights into the broader context of phenological responses to environmental changes.

## 1. Introduction

Plants have evolved characteristics that allow acclimation to the environment. This is particularly evident for reproductive events, which involve the entire development and release of pollen, the timing of which varies depending on interactions with the environment [1]. In addition, the timing of meiosis and, subsequently, fertilization varies along the inflow of development (evolution), making crops more resilient when exposed to transient stresses [2]. However, asynchronous development (asynchronous gametogenesis), more frequent in dioecious plants or in hermaphroditic plants with unisexual flowers, complicates the analysis of environmental impacts on yield and quality, compromising the selection of stress-tolerant crops. The significant impact of genotype, genotype–environment interactions, and asynchrony on reproductive development is very important. It has long been studied in cereals, then neglected due to conflicting research results [3]. The subject matter holds significant importance; hence, this study aims to explore its details for future advancements. Specifically, we investigate this phenomenon in an arboreal species like *Olea europaea* L. to contribute novel insights. Like most eukaryotes, meiosis is essential for sexual reproduction in plants, but it is strongly influenced by environmental stresses such as extreme temperatures. The potential reasons for the susceptibility of meiosis (e.g., during meiotic recombination) to abiotic stresses and its relationship with fertility have been extensively reviewed [2,4,5,6,7]. Microsporogenesis (formation of male gametes), gametogenesis (development of microspores), and maturation (formation of mature pollen) are a complex of very delicate activities, recognized as a key factor in determining the loss of yield in crops [8], especially in anemophilous pollinated species that are required to differentiate a large quantity of pollen [9]. During sporogenesis, the meiosis event is particularly vulnerable to environmental stress, significantly reducing the yield of cereals [8,10,11] and other crops [2,12,13].

Therefore, a thorough analysis of floral development is needed to accurately determine and analyze the meiotic stage to reveal the impact of abiotic stress [14]; this stage is crucial for the formation of haploid microspores and subsequent viable pollen grains [15].

Determining meiosis and the subsequent stages of pollen development in anemophilous plants often involves the destruction of young male inflorescences (as in dioecious plants) to dissect individual anthers. However, in catkin plants such as *Corylus*, *Alnus*, and *Betula*, morphological markers are commonly used to predict the stages of pollen development in a non-destructive manner. For example, catkin growth stages serve as reliable indicators to estimate the timing of meiosis [9,16]. A similar approach is employed for cereals, as demonstrated by Tottman [17] and Wang et al. [18]. In hermaphroditic plants like Olea, determining the timing of male meiosis requires careful manipulation of very young flower buds, meticulously removing the perules without damaging the delicate anthers.

In olive trees, climatic conditions, ranging from low winter temperatures to sudden spring warming and rainfall, significantly influence the development of reproductive structures, including flower formation and pollen grain production [19]. Moreover, cumulative rainfall during the flowering period can affect both the duration and the onset of pollination [20,21,22].

Notably, numerous studies highlight reproductive phenology as a valuable and sensitive indicator of the impacts of climate change [23,24,25]. However, the availability of extensive, long-term datasets required to identify significant phenological trends remains limited.

Airborne pollen data are a validated tool for the indirect assessment of flowering time [26,27,28,29]. It could be of great interest to consider adding to pollination monitoring a second, closely related micro-phenology observation, namely, the timing of male meiosis: why male meiosis is the determinant of pollination [30]. Introducing further knowledge into the complex phenomenon of flowering of a tree species that is strongly influenced by the environment in which it develops could provide further information for the correct identification of plant–environment relations. Furthermore, the time gaps between meiosis and anthesis is a physiological flower development period that could be decisive for pollination success, so it is certainly interesting to study these relationships. The study conducted goes in this direction, analyzing in a Mediterranean olive-growing area the pollination of olive trees by including, in the pollination database, the monitoring of the male meiosis event. The aim is to investigate a possible relationship between the dates of meiosis and the beginning and peak of pollination, as well as to identify the influence of meteorological variables, mainly temperatures and rain accumulations, on the date of meiosis and pollination.

## 2. Results

Different percentages of seasonal pollen concentration (SPIn) (5%, 10%, 20%, and 30%) were applied to determine the start of pollination (SP). SP calculated at 10% exhibited the least variability (i.e., the lowest standard deviation values) across years, so 10% (SP_10%) was identified as the optimal threshold.

**Figure 1:**
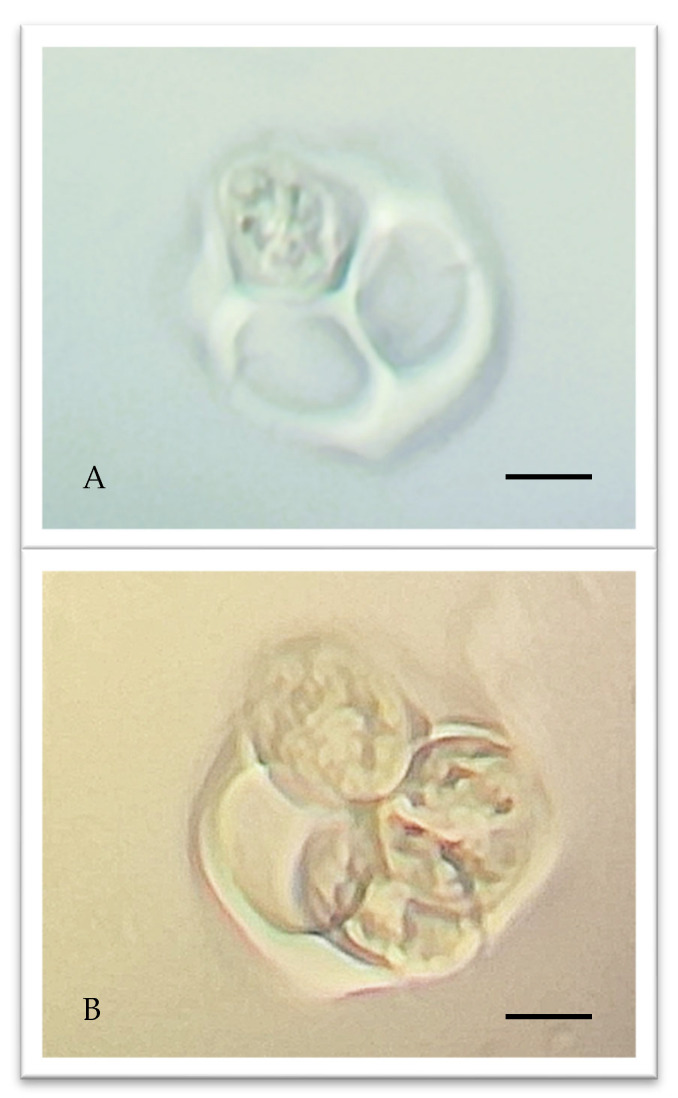
Tetrads of microspores in 2 cultivars of olive (*Olea europaea* L.) during the last male meiosis stage still in the callose envelope: Frantoio cv. (**A**); Leccino cv. (**B**). The unit of measurement equivalent to 5 microns is given.

The study of micro-phenological stages, focusing on male meiosis, was conducted using microscopic analyses. Figure 1 illustrates the tetrads of microspores, which are enclosed within the callose envelope, a clear indicator that male meiosis has just concluded. Shortly thereafter, cleavage occurs, releasing the microspores as monads that embark on their subsequent physiological maturation processes. On the observation day, most anthers showed tetrads, only a few were still in the premeiotic stage, and for a few others, cleavage had just occurred.

**Figure 2:**
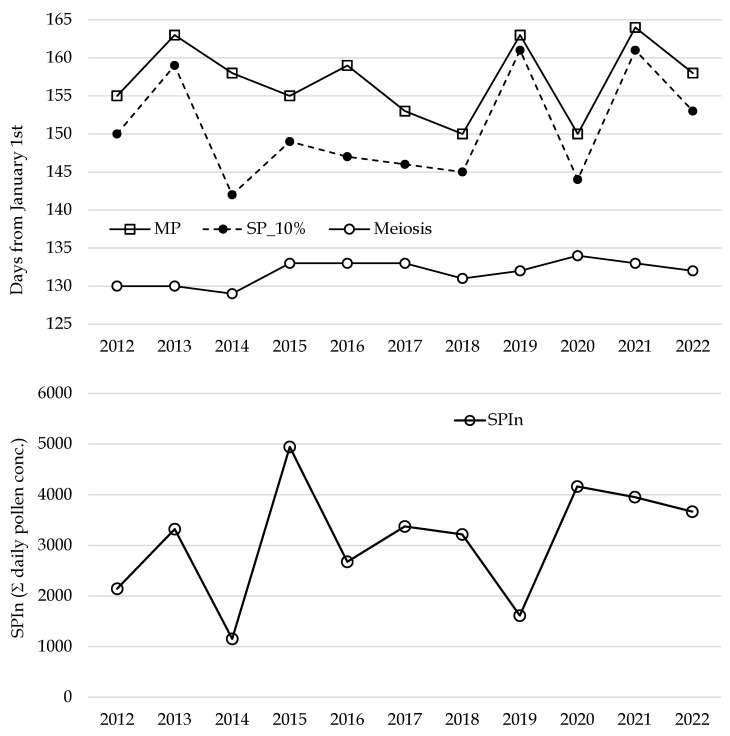
Phenological data (SP_10%, MP, meiosis, and SPIn) recorded in the study period (2012–2022).

This micro-phenological observation was conducted annually between 2012 and 2022, focusing on recording the dates of the meiotic phase (Figure 2). The results revealed remarkable consistency across more than a decade of observations: the meiotic phase occurred within a narrow 5-day window, during the first 10 days of May (129 to 133 days from January 1st, with a mean of 131 and a statistical mode of 132 days from January 1st). In contrast, the onset of pollination and peak (MP) showed greater variability, falling within a broader range of 10–15 days.

These results suggest that the influence of atmospheric variables (such as temperature and rainfall) on physiological processes preparing the pollination (like male meiosis) is distinct from their impact on mechanical phenomena, including anther dehiscence and pollen dispersal dynamics. Furthermore, this study highlighted notable heterogeneity in pollen concentrations throughout the years, ranging from a minimum of 1000 to a maximum of 5000 monitored pollen grains (Figure 2).

In terms of temperature accumulation, the most appropriate “base temperature” for calculating heat accumulation was identified as 7 °C, with a maximum threshold of 27 °C; in general, the starting date for calculating GDD, in the area under study, is January 1st [21,29]. The parameters used are in line with other research groups in olive growing, especially in the cooler Mediterranean area, which have identified the best “base temperature” and maximum temperature as being between 4.5 and 7 °C and between 26.3 and 31.5 °C [31].

The phenological interpretation of olive pollen monitoring from 2012 to 2022 is presented in Figure 2, which highlights key parameters: total pollen concentrations throughout the pollination period (SPIn); the dates (days from January 1st) marking the onset of pollination (SP); the dates of airborne pollen peak, MP; and the timing of male meiosis. These data provide valuable insights into the reproductive phenology of olives and their responses to environmental variables over the study period.

**Figure 3:**
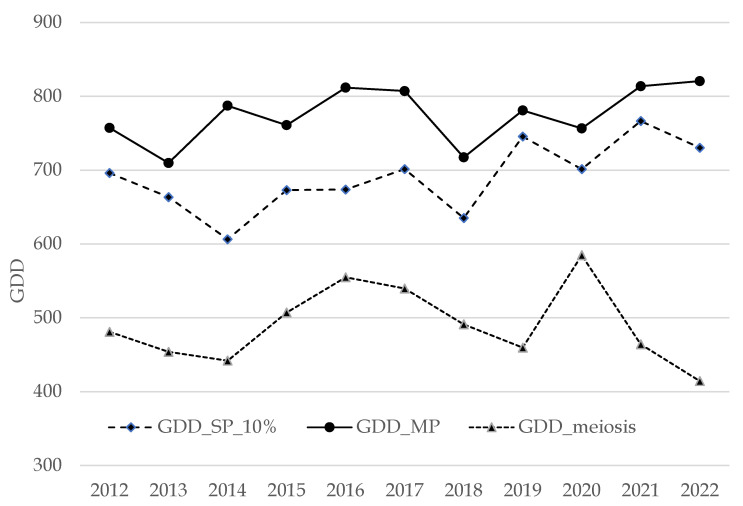
Yearly temperature summations (GDDs) calculated at the meiosis and pollination dates (SP_10%, MP), showing different amounts during the development of flower structures.

Figure 3 shows the yearly temperature summations (GDDs) calculated at the date of meiosis and pollination (SP and MP). The results demonstrate different temperature requirements during the development of flower structures.

Table 1 shows the statistical analyses carried out considering the annual dates of meiosis and flowering events. A strong correlation was found between SP and MP, showing a fast pollen release pattern, like a narrow Gaussian curve, as is typical in olive, but in recent decades, the curve showed an asymmetric trend to the left, i.e., with the peak (MP) close to the start of pollination (SP). The correlations between meiosis date and pollination date were not significant (r = −0.18 and −0.04). These events occur at least three weeks apart. The first event is undoubtedly the determinant of the subsequent ones, so they are closely related from a physiological point of view. The analytical non-correlation between the micro- and macro-phenological phases studied highlights the crucial role of external factors in the macro-phenological events.

Regarding the relationships between meiosis and environmental variables, the summation of temperature (GDDs) and rainfall amount calculated at the time of meiosis observation showed significant correlation values considering the study years (Table S1). In fact, the GDDs calculated at meiosis showed positive correlation values (r = 0.65), while with rainfall the correlation has negative values (r = −0.62), suggesting that a delay in meiosis may be correlated with higher temperature accumulation and lower rainfall. In the period for which meiosis data are available (2012–2022), a strong inverse correlation is observed between GDD accumulated at meiosis and the delta of GDD (∆ GDD) between meiosis and pollination, both early (r = −0.89) and maximum (r = −0.88). Higher GDD accumulation at meiosis results in lower GDD requirements for subsequent pollination initiation, and vice versa.

When evaluating the temperature and rainfall summations between the meiosis phase and pollination (Δ_GDD and Δ_Rain), a strong correlation was observed between Δ_GDD and SP_10%: r = 0.88; MP: r = 0.73. In contrast, rainfall summations (Δ_Rain) demonstrated no significant correlation with SP_10%: r = 0.35; MP: r = 0.28. This finding highlights the influence of temperature accumulations over rainfall in determining pollination events.

The flowering phase is closely correlated with temperature accumulations calculated from the start of the year, as shown in previous analyses using long-term historical data from 1982 to 2023 [21]. Furthermore, pollination appears to be influenced by the Δ_GDD values calculated specifically between meiosis and SP and MP, suggesting that GDD accumulation during this interval plays a crucial role.

Delayed pollination was associated with higher Δ_GDD values from meiosis, while lower accumulations correspond to earlier pollination. Meiosis, as the initiation point of pollen differentiation, marks the beginning of a critical period during which atmospheric variables directly affect the pollen maturation process.

Meiosis dates exhibit remarkable consistency across years (mean = 132.62; standard deviation = 0.86; coefficient of variation = 0.0062) compared to flowering dates. This consistency highlights the potential role of photoperiodic induction alongside annual climatic patterns in regulating meiosis. Conversely, SP and MP are more variable and are significantly influenced by the temperature. Notably, higher GDD accumulations at meiosis tend to result in earlier SP_10%: (r = −0.70). A strong positive correlation exists between SP_10% and the difference in GDD between meiosis (r = 0.88). Interestingly, this same variable (Δ_GDD_meiosis_SP_10%) shows a strong inverse correlation with GDD accumulation at meiosis (r = −0.89).

These relationships reveal that cooler spring conditions with lower GDD values at meiosis may increase the thermal requirements between meiosis and pollination, leading to a delayed flowering phase relative to average dates. This is further supported by significant negative correlations between SP_10% and MP and GDD values at meiosis, respectively (r = −0.70 and r = −0.55). These findings suggest the complex interplay between photoperiodic signals, temperature accumulation, and the timing of key reproductive phases in olive.

## 3. Discussion

Phenological observations in agriculture provide important information for farmers, and in addition to macro-phenological stages, further details can be obtained from micro-phenological stages [32,33]. In fact, in this research, the timing of male meiosis was studied as an observation of micro-phenology, detecting the process of pollen grain-forming (microgametogenesis) as a critical stage in sexual reproduction.

Micro-phenology observations showed remarkable consistency in the timing of male meiosis in olive trees, despite considerable climatic variation over the past decade. Anemophilous plants inherently have greater uncertainty about the success of sexual reproduction; therefore, male meiosis must be maximally protected from environmental stresses for the survival of the species; this explains the phenomenon of maladaptive phenological plasticity. The suggested phenomenon may refer to a situation where the timing (phenology) of reproductive events does not change in response to environmental conditions. In olive trees, this could mean that shifts in flowering period imposed by climate variations do not necessarily align with physiological conditions necessary for pollination and fertilization, compromising reproductive efficiency.

Our evidence suggests that male meiosis in olive is a physiological event also regulated by photoperiod (daylight within a day) as well as temperature forcing, as these plants have evolved mechanisms to minimize environmental stress on male meiosis, ensuring the production of viable pollen despite external fluctuations. In fact, photoperiod is a more stable environmental cue across years compared to temperature, which can vary unpredictably due to climate change. This reliance on day length ensures that key reproductive processes occur in a consistent seasonal window, rather than being disrupted by short-term temperature anomalies. However, if climate change alters not only temperature but also precipitation patterns, the photoperiod-driven reproductive schedule may become misaligned with optimal conditions for pollination and fruit set, reinforcing maladaptive phenological plasticity [34,35].

Nevertheless, air temperature plays a key role in successive macro-phenological expressions [31,36], especially in influencing the time interval (days) between meiosis and the start of pollination. Even micro-phenological processes are not exempt from atmospheric variability, considering that correlation analyses revealed that the timing of meiosis is influenced by both temperature sums and precipitation, but in opposite ways: temperature has a positive correlation, while precipitation has an inverse influence. This dual control defines the narrow time frame within which meiosis occurs.

Moreover, a more detailed analysis suggests that precipitation has a stronger influence on male meiosis than temperature. Increased precipitation advances meiosis, shortening the thermal accumulation period, while lower precipitation delays meiosis, extending the time required to meet thermal needs and resulting in higher GDD values [37]. This relationship signifies that GDD values for anthesis can be accurately estimated based on the date of meiosis. Importantly, meiosis, which is remarkably stable across years, allows for the reliable prediction of pollination dates with a reasonable margin of accuracy.

The observed maladaptive phenological plasticity of male meiosis sheds light on the resilience of the olive tree to the rising temperatures experienced in central Italy. According to Fornaciari et al. [21], olive can adapt to temperature increases by advancing pollination up to a maximum threshold of 25 May, beyond which no further advancement is possible. Comparable observations have been reported in other woody species, where climate warming has led to an advancement of spring phenological events. However, species with a strong sensitivity to photoperiod are unlikely to fully adjust their developmental timing in response to continued warming. This limitation arises because photoperiodic cues—unlike temperature—remain constant over time and thus increasingly constrain the phenological plasticity of such species under changing climatic conditions [38].

This finding is supported by the present research, since the timing of male meiosis requires a specific interval time for pollen development and maturation. The phenological plasticity of olive pollination, while generally considered adaptable, may under certain conditions shift toward a form of phenological rigidity that could reduce the plant’s responsiveness to temperature fluctuations. This potential trait suggests a degree of resilience to climate variability in olive and may contribute to its capacity to persist under changing environmental conditions.

## 4. Materials and Methods

### 4.1. Study Area

This study utilized aerobiological data obtained from pollen monitoring in Perugia (43°6′ N, 12°23′ E, 450 m above sea level) in Umbria, central Italy. Olive cultivation accounts for about 9% of the region’s land use (2020 Italian agricultural census) with 26,552 ha of olive groves (https://www.istat.it/it/archivio/273753 accessed on 12 July 2025), primarily concentrated on hills between 150 and 500 m above sea level. The Apennines shield this area from cold eastern cyclones.

Umbria’ extra virgin olive oil production received EU Protected Designation of Origin certification (PDO) meeting specific local cultivar requirements being constituted by 5 geographical mentions depending on the cultivars of olive trees used. The majority of olive cultivars present around the pollen trap were Frantoio cv. and Leccino cv. Airborne pollen data were collected following the specifics in CEN/TS 16868, 2019—Sampling and analysis of airborne pollen and fungal spores for aerobiological monitoring-UNI, Italian National Unification: Milano, Italy 2019 [39].

**Figure 4:**
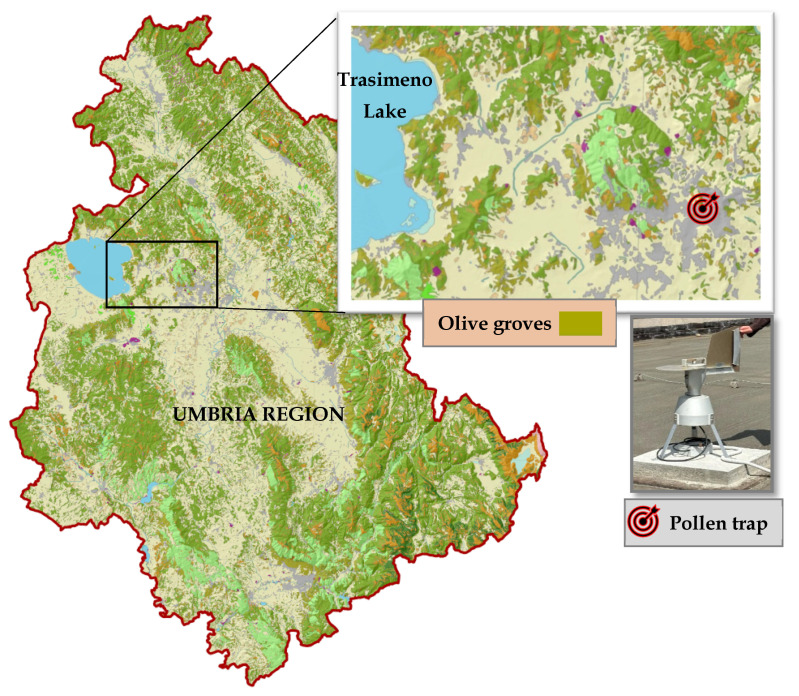
Pollen trap location inside the olive grove areas in Umbria Region.

The device used was a Hirst-type volumetric pollen trap. In aerobiological monitoring by volumetric spore trapping, airborne biological particles (pollen and spores) are collected on sticky surfaces (tapes) transformed into samples (slides) corresponding to 24 h of air monitoring. Examination of these surfaces involves counting and identification of particles under an optical microscope according to the cited standardized method. We applied the most stringent requirements for data reliability by examining, on each sample, more than 15% of the total surface area. EN 16868, 2019 standards set the threshold at 10% or higher, for environments with medium-to-high pollen density. The device was located in a periurban area surrounded by olive groves. This allows for daily quantification of pollen concentration and the determination of the pollen season’s start, maximum, end, and intensity (Figure 4).

### 4.2. Aerobiological and Meteorological Data

The study was performed using an 11-year database (2012–2022) of airborne pollen and environmental variables provided by a pollen monitoring and meteorological station located in Perugia. The olive pollen sampling activities were carried out using the volumetric method, which is based on capturing the pollen and other biological particles present in the air (International Association for Aerobiology 2011). The Hirst-type volumetric pollen trap (VPPS 2000, Lanzoni Ltd., Bologna, Italy) was in a windy site of Perugia city for the detection of the pollen from a wide olive-growing area. This kind of sampling reflects the anthesis phenomenon, reducing the subjectivity in the interpretation of the pollination period using field observations ([28]. The daily pollen capture was expressed in olive pollen grains/m^3^ of air according to the scientific literature.

Three phenological features were calculated for each year of pollen monitoring: the total pollen concentrations captured during the entire pollen emission period (Σ daily pollen concentrations = Seasonal Pollen Index, SPIn); the date (days from January 1st) related to the beginning of significant olive pollen quantity present in the atmosphere (start of pollination, SP); and the date of the maximum daily pollen concentration (maximum pollination, MP).

To establish year by year the start of pollination dates, different percentages of the total pollen concentration (5–10–20–30% SPIn) were calculated whose attainment date was set as SP in all monitoring years. The difficulty of correctly establishing the start of pollination is caused by the large geographical capacity of the pollen trap to intercept pollen grains and consequently by the false-start effect due to sporadic olive trees beginning the process of anthesis. To measure the initial pollen concentration in a different way, as reported in other studies [40,41], one can identify the moment when most of the olive trees are involved in the full flowering phenomenon, which thus indirectly represents the massive flowering in the different olive orchards around the pollen trap.

The daily meteorological data were provided by the official weather station of the Umbria Region, the regional hydrographic service. Moreover, the temperature amounts (GDDs) for the different flowering dates were calculated to highlight the presence of forcing phenomena related to biological reproductive structure development.

### 4.3. GDD Calculations

The forcing units were expressed as growing degree days (GDDs) and were calculated using the method proposed by Arnold [42], based on the maximum and minimum daily temperatures. This model assumes a temperature base below which biothermic accumulation stops or is reduced to its lowest terms. To determine the most appropriate base temperature for the heat accumulation, 6–7–8–9–10 °C were tested while maximum temperatures of 26–27–28 °C were tested, with January1st (t) used as the forcing start date. The GDDs were calculated for the SP and MP dates for each study year. The root mean square errors (RMSEs) were calculated. The most accurate forcing start date and the best threshold temperature were selected for each pollination date, according to the lowest mean RMSEs over the study period.

### 4.4. Male Meiosis Phase Identification

In addition, a micro-phenological study provides the annual dates of male meiosis that occurred in the olive groves located around the study area. This study was carried out from 2012 to 2022 in two of the most commonly used olive cultivars (Frantoio cv. and Leccino cv.) of the cited monitoring area.

To identify the phenological phase representative of the buds’ last reproductive development, the external morphology of young flower buds was closely examined in some olive groves representative of the aerobiological monitoring area.

**Figure 5:**
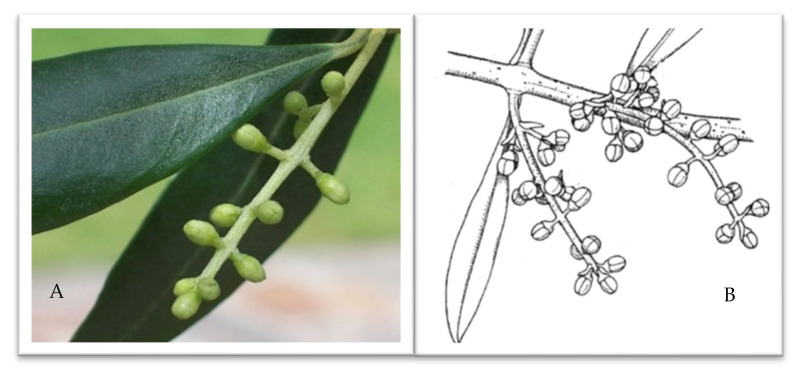
External morphology of young flower buds (**A**) during macro-phenological observation, magnification 40×; BBCH 57 phase corresponding to the reproductive development phase (**B**).

When the majority of the plants showed slender buds, ovoid in shape, displaying a uniform grass-green color throughout the inflorescence (Figure 5A), the BBCH 57 phase —“The corolla, green-colored, is longer than calyx” [43]—was attributed to the whole olive grove (Figure 5B). As the buds matured, their morphology evolved, becoming increasingly globular with a pale green hue and a whitish tip. When olive inflorescences reached the BBCH 57 phase, they were considered suitable for micro-phenological analyses aimed at studying male meiosis; 10 buds from 10 trees of the more representative olive cultivars of the studied area at different altitudes and sun exposures were analyzed. On the samples, the perules were gently pulled off under a Leica L2 stereomicroscope (Leica Microsystems, Wetzlar, Germany) equipped with Nikon D7500 (Nikon Europe B.V. Amstelveen, The Netherlands). Then, the anthers were removed, placed on a microscope slide, squeezed to release the contents, and finally observed under a LEICA DM 4000B light microscope (Leica Microsystems, Wetzlar, Germany).

## 5. Conclusions

The results of this research underscore the fundamental role of micro-phenological observations in enhancing our understanding of macro-phenological manifestations. By bridging these two scales, this study introduces an innovative approach within the field of eco-agro-phenology. One of the major contributions of this work is its potential to refine the calculation of thermal requirements for anthesis in olive trees. The identification of the precise moment of male meiosis provides a starting point for determining when the plant begins to calculate its thermal needs to initiate the reproductive phase.

Moreover, this research sheds light on key aspects of the anthesis phenophase in olive trees, highlighting a novel connection between the species widely acknowledged phenological plasticity and the micro-phenological rigidity of male meiosis. This finding suggests that the response of olives, and likely other arboreal species, to climate change is not straightforward but rather a delicate balance. It reflects the dual necessity of supporting the maturation of microgametophytes while synchronizing fertilization with optimal climatic conditions.

The insights gained here pave the way for further investigations into the effects of photoperiod and thermo-pluviometric factors on male meiosis and flowering in olive trees cultivated in geographical regions with different photoperiodic conditions.

Additionally, this research highlights the need to revaluate the controversial role of precipitation in influencing reproductive phenology. Beyond olives, the study opens up exciting opportunities to explore the phenology of other anemophilous agricultural species, such as hazelnut species, to examine the interaction between meteorological variables, photoperiod, pollination, and productivity within the context of climate change. This work not only highlights the potential importance of phenological processes but also offers practical implications for crop management and adaptation strategies in an era of global environmental shifts.

## Figures and Tables

**Table 1:** Correlation analysis considering annual values from 2012 to 2022 (* *p* < 0.05; ** *p* < 0.01).

	Meiosis	SP_10%	MP	GDD_Meiosis
Meiosis	1.00			
SP_10%	−0.18	1.00		
MP	−0.04	0.91	1.00	
GDD_Meiosis	0.65 *	−0.70 **	−0.55	1.00
GDD_SP_10%	0.24	0.83 **	0.75	−0.46
GDD_MP	0.31	0.43	0.65 *	−0.25
Δ_GDD_Meiosis_SP_10%	−0.31	0.88 **	0.74 **	−0.89 **
Δ_GDD_Meiosis_MP	−0.34	0.74 **	0.73 **	−0.88 **
Σ_Rain_Meiosis	−0.62 *	−0.08	0.02	−0.05
Σ_Rain_SP_10%	−0.62	0.06	0.14	−0.08
Σ_Rain_MP	−0.54	−0.01	0.12	0.02
Δ_Rain_Meiosis_SP_10%	−0.38	0.35	0.36	−0.12
Δ_Rain_Meiosis_MP	−0.16	0.13	0.28	0.15

## Data Availability

The data that support the findings of this study are available from the corresponding author, Fabio Orlandi, upon reasonable request.

## Supplementary Materials

The following supporting information can be downloaded at: https://www.mdpi.com/article/10.3390/plants14162522/s1, Table S1: Yearly temperature summations (GDD) calculated at the date of meiosis and at different starts and maximum of pollination (SP_5-10-20-30%, MP). Moreover, the GDD from meiosis to SP_10% and to MP are shown.
