# Supplementary material for: Exploring Reproductive Timing in Olive Tree: Male Meiosis and Anthesis Events
Source: Plants (Basel). 2025 Aug 13;14(16):2522. doi: 10.3390/plants14162522 (PMC12389715; doi:10.3390/plants14162522)
Supplement: Supplementary file 1 [file plants-14-02522-s001.zip › plants-3795557-supplementary.pdf]

Supplementary material - Table S1

Yearly temperature summations (GDD) calculated at the date of meiosis and at different starts and maximum of pollination (SP_5-10-20-30%, MP). Moreover, the GDD from meiosis to SP_10% and to MP are shown.

Years	GDD_ SP_5%	GDD_ SP_10%	GDD_ SP_20%	GDD_ SP_30%	GDD_MP	GDD_ meiosis	Δ_GDD_ Meiosis_SP_10%	Δ_GDD_ Meiosis_MP
2012	666.03	696.10	709.85	721.37	757.29	481.30	214.80	275.99
2013	663.46	663.46	696.50	696.50	709.76	453.70	209.76	256.06
2014	591.38	606.33	697.39	730.15	787.31	442.20	164.13	345.11
2015	660.51	672.95	698.76	713.23	760.98	507.14	165.81	253.84
2016	647.10	673.85	689.70	711.27	811.77	554.88	118.97	256.89
2017	587.88	701.45	745.33	760.80	807.17	539.70	161.75	267.47
2018	620.46	635.11	651.16	667.40	717.37	491.17	143.95	226.20
2019	727.18	745.61	763.34	763.34	780.89	459.61	286.01	321.29
2020	672.88	701.33	724.75	733.44	756.54	584.77	116.56	171.77
2021	737.45	766.56	766.56	780.90	813.66	464.16	302.39	349.49
2022	680.96	730.30	768.15	786.62	820.59	414.37	315.93	406.22
